# A non-human primate model of acute liver failure suitable for testing liver support systems

**DOI:** 10.3389/fmed.2022.964448

**Published:** 2022-09-30

**Authors:** Ranjeet S. Kalsi, Alina Ostrowska, Adam Olson, Mubina Quader, Melvin Deutsch, Norma J. Arbujas-Silva, Jen Symmonds, Alejandro Soto-Gutierrez, John J. Crowley, Miguel Reyes-Mugica, Giselle Sanchez-Guerrero, Hartmut Jaeschke, Bruce P. Amiot, Marilia Cascalho, Scott L. Nyberg, Jeffrey L. Platt, Edgar N. Tafaleng, Ira J. Fox

**Affiliations:** ^1^Department of Surgery, University of Pittsburgh School of Medicine, Pittsburgh, PA, United States; ^2^Department of Pathology, University of Pittsburgh School of Medicine, Pittsburgh, PA, United States; ^3^Pittsburgh Liver Research Center, University of Pittsburgh, Pittsburgh, PA, United States; ^4^Department of Radiation Oncology, University of Pittsburgh School of Medicine, Pittsburgh, PA, United States; ^5^McGowan Institute for Regenerative Medicine, Pittsburgh, PA, United States; ^6^Division of Vascular and Interventional Radiology, Children’s Hospital of Pittsburgh of UPMC, Pittsburgh, PA, United States; ^7^Department of Pathology, Children’s Hospital of Pittsburgh of UPMC, Pittsburgh, PA, United States; ^8^Department of Pharmacology, Toxicology and Therapeutics, The University of Kansas Medical Center, Kansas City, KS, United States; ^9^Department of Surgery, Mayo Clinic, Rochester, MN, United States; ^10^Departments of Surgery and Microbiology and Immunology, University of Michigan, Ann Arbor, MI, United States

**Keywords:** acute liver failure (ALF), non-human primates (NHPs), liver-directed radiation therapy, hepatic ischemia-reperfusion injury, xenogeneic hepatocyte transplantation

## Abstract

Acute hepatic failure is associated with high morbidity and mortality for which the only definitive therapy is liver transplantation. Some fraction of those who undergo emergency transplantation have been shown to recover native liver function when transplanted with an auxiliary hepatic graft that leaves part of the native liver intact. Thus, transplantation could have been averted with the development and use of some form of hepatic support. The costs of developing and testing liver support systems could be dramatically reduced by the availability of a reliable large animal model of hepatic failure with a large therapeutic window that allows the assessment of efficacy and timing of intervention. Non-lethal forms of hepatic injury were examined in combination with liver-directed radiation in non-human primates (NHPs) to develop a model of acute hepatic failure that mimics the human condition. Porcine hepatocyte transplantation was then tested as a potential therapy for acute hepatic failure. After liver-directed radiation therapy, delivery of a non-lethal hepatic ischemia-reperfusion injury reliably and rapidly generated liver failure providing conditions that can enable pre-clinical testing of liver support or replacement therapies. Unfortunately, in preliminary studies, low hepatocyte engraftment and over-immune suppression interfered with the ability to assess the efficacy of transplanted porcine hepatocytes in the model. A model of acute liver failure in NHPs was created that recapitulates the pathophysiology and pathology of the clinical condition, does so with reasonably predictable kinetics, and results in 100% mortality. The model allowed preliminary testing of xenogeneic hepatocyte transplantation as a potential therapy.

## Introduction

The liver is a complex organ whose function is life sustaining and critical for such processes as protein synthesis, detoxification and metabolic regulation (1). As a result, liver failure is associated with very high morbidity and mortality, requiring complex medical interventions for metabolic abnormalities, coagulopathy, encephalopathy, and in the case of acute, fulminant hepatic failure, management can be further complicated by hypotension, hypoglycemia, increased intracranial pressure, multi-organ system failure, and sepsis (2). There are approximately 2,000 cases of acute liver failure that occur in the United States every year (3).

Recent therapeutic advances, especially in liver transplantation, have been transformative for patients affected by liver disease. Clinicians can approximate the extent of disease and risk of mortality for patients with cirrhosis and end-stage chronic liver failure by combining physical exam findings and laboratory studies by calculating Model for End-Stage Liver Disease (MELD) and Child-Pugh scores (4). However, in patients with acute liver failure (ALF), individual clinical and laboratory parameters do not always predict survival from non-survival (5–7). Nevertheless, because of advances in transplantation, mortality from ALF has dramatically improved (8). The Acute Liver Failure Study Group (USA) reports that approximately 45% of patients with severe acute liver failure recover liver function spontaneously, 25% undergo liver transplantation, and 30% expire, including approximately 30% of those transplanted (9). Because determining who will survive without transplantation is so difficult, it is known that some fraction of those who undergo emergency liver transplantation would have recovered without it. Thus, the increasing success in managing acute liver failure cloaks the particularly sad and frustrating fact that some fraction of those who undergo orthotopic liver transplantation for acute liver failure might nevertheless have spontaneously recovered liver function over a period of weeks to months if transplantation could have been averted.

Ideal management of acute liver failure would avert liver transplantation when recovery of the liver is possible, and when recovery does not occur, enable survival until a liver transplant can be performed. Development of such support has been a research priority in hepatology for decades. Unfortunately, development of liver support devices has been hindered by the low incidence of severe acute failure, the diverse clinical presentation, and by unpredictable outcomes. While liver support devices of various types have been developed, trials in cohorts with ALF conducted to date have generated inconclusive results, and their use failed to significantly increase survival or avert transplantation (10–13). The results do not suggest the devices were ineffective but instead confirm that an impossibly large number of subjects would need to be enrolled to overcome the confounding impact of the vast range of baseline indices of liver function and outcomes of ALF. Thus, the vast range of manifestations of acute liver failure has made it nearly impossible to reach conclusions regarding efficacy and to identify patients who might benefit.

To date, many thousands of patients have been treated with these devices. Artificial liver support systems can replace liver detoxification functions to a limited degree and correct some biochemical parameters; however, clinical data on improved survival is lacking (14). Actual replacement of liver function is thought to require development of bioartificial systems, which entails complex cell cultures and flow dynamics and, in turn, very high production costs (14, 15). Unfortunately, the true mechanisms by which hepatocytes fail and the consequences of their failing are not fully understood, and there is no fully workable model of hepatic failure that recapitulates what occurs in man except toxin-induced hepatic decompensation (16). The costs of development and the expense of running a clinical trial could be dramatically reduced by the availability of a reliable large animal model of hepatic failure that included a large therapeutic window that allows testing the efficacy and timing of intervention.

**FIGURE 1:**
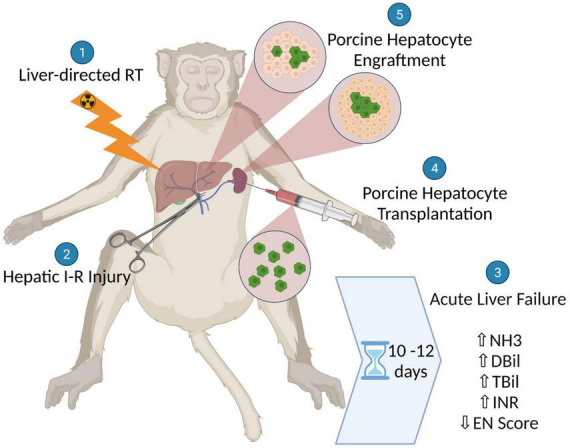
Schematic illustration showing the generation of a non-human primate model for acute liver failure (ALF) using liver-directed radiation therapy followed by hepatic ischemic-reperfusion injury and the subsequent assessment of xenogeneic hepatocyte transplantation as a potential therapy for ALF.

We created a model of severe ALF in non-human primates (NHPs) that recapitulates the pathophysiology and pathology of the clinical condition, does so with reasonably predictable kinetics, and results in 100% mortality. We show that after liver-directed radiation therapy (RT), the delivery of non-lethal hepatic ischemia-reperfusion (I-R) injury reliably and rapidly generates life-threatening manifestations of ALF that in turn provide conditions that can enable pre-clinical testing of liver support or replacement therapies. Indeed, we show that ALF induced in this way allowed preliminary testing of xenogeneic hepatocyte transplantation as a potential therapy (Figure 1).

## Materials and methods

### Non-human primates and central IV placement

Male cynomolgus macaques (*Macaca fascicularis*) weighing between 5 and 9 kg were used for these studies. The animals were kept at the John G. Rangos Sr. Research Center, UPMC Children’s Hospital of Pittsburgh. All NHPs were kept in a state-of-the-art facility with automatic 12-h light and 12-h dark cycles with lights on from 7 a.m. to 7 p.m. Water was readily available to the NHPs and their diet consisted of biscuits, fruits, vegetables, and forage mix. Prior to surgery, the NHPs required acclimatization to a jacket and tether system for 2 weeks. Immediately prior to surgery, NHPs were sedated using ketamine (5–10 mg/kg) and then underwent endotracheal intubation with subsequent administration of inhalational anesthesia in the form of isoflurane. A long-dwelling single lumen central venous catheter was inserted into the internal jugular vein. The catheter was tunneled subcutaneously to exit through the skin midway between the ipsilateral tip of the scapula and the vertebral spinous process. The jacket and tether system was used to protect the indwelling catheter and facilitate access. The central venous catheter is important for managing possible post-operative complications, including dehydration, electrolyte imbalance, and eventual liver failure, sampling blood, and administering medications. Table 1 provides a summary of procedures and findings in each animal.

**TABLE 1:** Summary of procedures and findings in non-human primates.

NHP ID	Induction of ALF after liver-directed RT	Transplant after induction of ALF
M001	NHP received a starting dose of 0.1 mL/kg of 40% CCl_4_ by SQ injection but experienced respiratory distress and expired 5 min after administration.	N/A
M002	NHP received increasing doses of 0.02–0.8 mL/kg of 40% CCl_4_ by SQ injection with no significant increases in ALT levels (30–50 IU/L). He received a higher dose of 1.8 mL/kg but experienced respiratory distress and expired during administration.	N/A
M003	NHP received increasing doses of 0.002–0.384 mL/kg of 40% CCl_4_ by oral gavage with no significant increases in ALT levels (91–152 IU/L).	N/A
M004	NHP received a starting dose of 0.032 mL/kg of 40% CCl_4_ by oral gavage that led to ALT levels of 1,131 IU/L. He received a higher dose of 0.064 mL/kg by oral gavage that led to lower ALT levels of 345 IU/L. He progressively showed abnormal NH3, total and direct bilirubin, INR, and EN score.	N/A
M005	NHP underwent 80 min I-R injury. He progressively showed abnormal ammonia level (NH3), total and direct bilirubin, INR, and encephalopathy score.	N/A
M006	NHP underwent 90 min I-R injury. He progressively showed abnormal NH3, total and direct bilirubin, INR, and EN score.	N/A
M007	NHP underwent 90 min I-R injury followed by pig hepatocyte transplant on the same day.	NHP was transplanted with 300 million porcine hepatocytes on the same day as I-R injury. Hepatic function appears to stabilize post-transplant but the NHP developed renal failure and was euthanized 7 days after I-R injury and transplant.
M008	NHP underwent 90 min I-R injury. He progressively showed abnormal NH3, total and direct bilirubin, INR, and EN score.	NHP was transplanted with 600 million porcine hepatocytes 83 days after I-R injury. Hepatic function appeared to stabilize post-transplant but the NHP expired of hepatic failure 12 days after transplant.
M009	NHP underwent 90 min I-R injury. He progressively showed abnormal NH3, total and direct bilirubin, INR, and EN score.	NHP was transplanted with 1 billion porcine hepatocytes 14 days after I-R injury. Hepatic function appeared to stabilize post-transplant but the NHP expired of CMV pneumonitis 14 days after transplant.

### Liver-directed radiation therapy

All primates were anesthetized using isoflurane and immobilized in the supine position with whole-body Vac-Lok cushions (CIVCO, Kalona, IA) during pre-intervention CT and radiation therapy (RT) with their arms above their head. Pre-intervention CT scans were obtained, procuring 2.5 mm slices with IV contrast from the level of the carina to the roof of the acetabulum. Radiation oncology marked the location of the isocenter on the skin (AP and lateral). Next, scanned CT images were transferred to an Eclipse V15.6 treatment planning system (Varian Medical System, Palo Alto, California), and whole liver (PTV) and organs at risk – duodenum, esophagus, small bowel, and spinal cord were contoured by the radiation oncologist. The treatment plans were then generated using 11–12 coplanar IMRT fields. The prescribed dose for the whole liver (PTV) was 35Gy delivered in 5 fractions. On the day of treatment, the skin marks and SSD at the isocenter obtained from the treatment plan were used for the initial set-up. After correctly positioning the primate at the isocenter, CBCT (cone-beam computed radiotherapy) images were acquired. Image co-registration process (for all CBCT registrations) was done by the radiation oncologist, and co-registrations were used to correct the treatment position, ensuring proper positioning in all three cardinal planes. Each primate received whole liver stereotactic body radiotherapy treatments (SBRT) using a 6MV beam from a CLINAC 23IX Varian accelerator (Varian Medical System, Palo Alto, California) CBCT image guidance.

### Induction of acute liver failure by carbon tetrachloride (CCl_4_) treatment after radiation therapy

For treatment of NHPs with CCl_4_ by subcutaneous injection, a 40% (v/v) solution of CCl_4_ (Sigma, St. Louis, MO) was prepared by diluting CCl_4_ in olive oil. An initial dose of 0.2 mL of 40% CCl_4_ solution per kg body weight was used for each naïve NHP with subsequent doses increased based on elevations in alanine aminotransferase (ALT) levels measured 2 days after every CCl_4_ treatment. This dose-escalation approach was followed for each NHP until an effective dose that led to an ALT level of 300–1,000 IU/L was attained. Because the irradiated liver is potentially more susceptible to CCl_4_–induced liver injury, the initial CCl_4_ dose used for each NHP after liver-directed radiation therapy was lower than the effective dose determined before radiation treatment. However, increasing CCl_4_ doses did not consistently generate higher ALTs, and 2 out of 3 animals developed bronchospasm and diffuse pulmonary hemorrhage to subcutaneously administered CCl_4_ after hepatic radiation. The route of CCl_4_ administration was therefore modified for subsequent NHPs because pulmonary side effects were never observed when NHPs were given CCl_4_ by oral gavage.

For treatment of NHPs with CCl_4_ by oral gavage, a 10% (v/v) solution of CCl_4_ was prepared by diluting CCl_4_ in olive oil. A starting dose of 0.002 mL of 10% CCl_4_ solution per kg body weight was used for each NHP with subsequent doses increased based on elevations in alanine aminotransferase (ALT) levels measured 2 days after every CCl_4_ treatment. This dose-escalation approach was followed for each NHP until an ALT level of 300–1,000 IU/L was attained. Because CCl_4_ treatment proved to be an unreliable method for inducing ALF after radiation therapy, I-R injury was assessed as a potential replacement for CCl_4_ treatment.

### Induction of acute liver failure by surgically induced transient ischemia-reperfusion injury after radiation therapy

Through a midline laparotomy, the portal triad, which contains the extrahepatic segments of the hepatic portal vein, hepatic artery proper, and the common bile duct, was identified and circumferentially dissected to ensure that there were no accessory or replaced arteries to the liver. The NHP was then heparinized (100 U/kg) and the portal triad was occluded using multiple bull dog clamps. Ischemia to the liver was sustained for between 80 and 150 min. Upon completion of the designated ischemia time, the clamps were removed, and the area was evaluated for any potential iatrogenic injuries or areas of bleeding. The abdomen and skin were closed in a standard fashion and a clean jacket and tether system was then applied.

### Scoring for hepatic encephalopathy

The level of hepatic encephalopathy was determined based on observations or tests for appetite, attention, neurologic condition, and strength following a rating scale (Supplementary Table 1). Encephalopathy scores (EN score) were then calculated by combining the points for each of the four subtests.

## Results

### High dextrose-containing parenteral nutrition or CCL_4_ produce irreversible acute liver failure following liver-directed radiation

We previously reported that fulminant liver failure can be induced in monkeys by administration of liver-directed radiation followed by infusion of high dextrose-containing parenteral nutrition (TPN) (17). High-dose radiation inflicts injury and interferes with regeneration, while the high dextrose TPN infusion induces a non-specific injury to hepatocytes. The histology of the liver and clinical manifestations of acute liver failure induced in this way in monkeys resembles characteristics of acute liver failure in humans (17). However, consistent with manifestations of acute liver failure in humans, monkeys exhibited marked variation in the kinetics of development of liver failure. Although our method could reliably recapitulate the clinical presentation of acute liver failure, the variation in timing would hinder if not preclude use for testing therapeutics, such as liver assist devices or transplantation. We therefore sought to modify the model to generate more uniform and predictable injury.

We first attempted to control the timing of liver failure by administering CCl_4_, which reliably induces liver failure in rodents at least in part by known mechanisms (18). Unfortunately, we observed numerous unanticipated problems with this approach. Animals were given escalating doses of CCl_4_ before receiving radiation therapy so that a reproducible liver injury could be anticipated post radiation, aiming for an ALT level between 300 and 1,000 IU/mL at 48 h following exposure. Unfortunately, animals experienced pulmonary distress and expired from subcutaneously administered CCl_4_ after hepatic radiation (Table 1, M001 and M002). CCl_4_ treatment by oral gavage did not induce bronchospasm and diffuse pulmonary hemorrhage, but the treatment generated even more inconsistent levels of hepatic injury (Table 1, M003 and M004). Escalating doses of CCl_4_ did not always generate higher ALTs, and, following liver-directed radiation therapy, the selected dose of CCl_4_ often no longer produced the desired level of liver injury.

### Homogeneity of acute liver failure generated by liver-directed radiation followed by hepatic ischemia-reperfusion (I-R)

Reasoning that genetic differences were less likely to affect the degree of hepatic injury mediated by ischemia, we next tested whether hepatic ischemia superimposed on irradiation could generate acute liver failure and do so with greater homogeneity than RT plus TPN or CCl_4_. To induce liver ischemia, the portal vein and hepatic artery were occluded for 80–150 min. After induction of ischemia, the vessels were re-perfused and the monkeys were carefully monitored during recovery and subsequent course.

**FIGURE 2:**
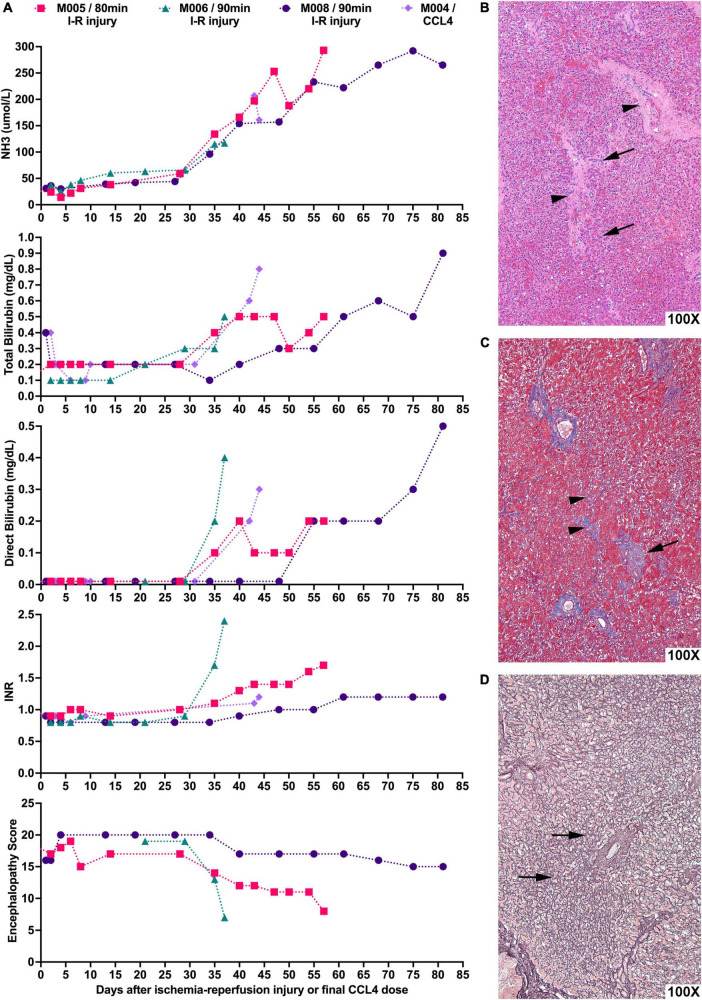
Liver-directed radiation therapy in combination with hepatic ischemic-reperfusion leads to severe liver failure requiring euthanasia with histological hallmarks consistent with acute liver failure. **(A)** NHPs underwent liver-directed radiation therapy and, after 2 weeks, were subjected to 80–90 min of hepatic I-R injury (day 0). NH3, total bilirubin, direct bilirubin, and INR levels as well as encephalopathy scores of NHPs were recorded. Animals developed severe liver failure requiring euthanasia 10–12 days after key indicators of hepatic function begin to show abnormal levels. M004 is included for comparison of CCl_4_-induced ALF with the day of last CCl_4_ dose set as day 0. **(B)** Representative photomicrograph of H&E stained liver sample showing extensive loss of hepatocytes with the collapse of the parenchyma leading to the approximation of portal tracts (arrowheads). Entire rows of hepatocytes have disappeared, leaving only a fraction of the original cells (arrows). **(C)** Representative photomicrograph of Masson’s trichrome staining of liver sample highlighting in blue the periportal collagen (arrow) and the interstitial fibrosis (arrowheads). **(D)** Representative reticulin staining of liver tissue showing that the fiber meshwork that normally lines each row of hepatocytes is now collapsed (arrows) as the intervening hepatocytes have died and disappeared.

As anticipated, I-R induced liver injury varied with dose, i.e., duration of ischemia, but manifested with greater homogeneity than metabolic insults. Ischemia for more than 120 min induced severe hepatic necrosis within hours of injury manifested by an ALT > 3,000 IU/L, NH3 > 300 μmol/L, and INR > 2 and death from hyperkalemia. Ischemia for 80–90 min led to relatively uniform liver injury with peak ALT levels of 327–849 IU/L 48 h after I-R induced liver injury. After that time, the ALT decreased and no signs of liver failure were evident for several weeks, but progressive liver dysfunction ensued leading to manifestation of severe hepatic failure with encephalopathy. Figure 2A shows the course of hepatic failure in three animals (M005, M006, and M008) treated with liver irradiation and hepatic ischemia, and in one animal (M004) treated with liver irradiation and CCl_4_ by gavage, who developed an ALT of 345 IU/L 48 h after exposure to last CCl_4_ dose. Indicators of hepatic function [ammonia level (NH3), total and direct bilirubin, INR, and encephalopathy score (EN score, normal EN score: 20)] became abnormal, and euthanasia was ultimately required. An increase in the ammonia, direct bilirubin, INR, and encephalopathy predicted severe liver failure requiring euthanasia within 10–12 days. Histology consistent with acute liver failure, including necrosis, loss of hepatocytes, and parenchymal collapse, was present in each liver (Figures 2B–D).

Several studies have reported unique cytokine profiles during the development of pediatric ALF and drug-induced liver injury (19–21). We therefore analyzed the serum cytokine levels in NHPs before and after treatment with liver-directed radiation and hepatic ischemia to determine if a similar trend was present in this NHP model of ALF. In contrast to clinical indicators of hepatic function which progressively deteriorated as the NHPs approached ALF, there was no consistent trend in the serum cytokine levels in this NHP model of ALF (Supplementary Figure 1).

Animals treated with this regimen can experience hemodynamic changes, dehydration, and electrolyte disturbances after I-R induced liver injury. Careful monitoring of vital signs, urine output, and chemistries in the preoperative and operative period was essential. As a result of low central venous pressures, two animals developed severe hemodynamic instability, renal failure, and neurologic abnormalities immediately following transient hepatic ischemia and required euthanasia.

### Hepatocyte xenotransplantation for acute liver failure

Having established that ALF could be reliably induced in randomly bred monkeys, we asked whether homogeneity would allow testing of a therapeutic strategy. We previously explored hepatocyte transplantation in rodent models of chronic liver failure (22) and others have shown that hepatocyte transplantation could rescue rodents with ALF (23, 24). However, the potential use of hepatocyte transplantation in a pre-clinical NHP model of ALF was never explored in part because variability of manifestations and course would make it difficult or impossible to evaluate outcomes, as discussed above. Thus, we exploited the homogeneous features of the model of ALF to undertake a pre-clinical test of hepatocyte xenotransplantation in ALF.

Three monkeys were treated with RT and I-R to induce ALF as described above. Porcine hepatocytes were isolated, and between 300 million to 1 billion hepatocytes were transplanted into each animal by direct injection into the spleen as we have previously published (Supplementary materials and methods) (25). Because we were interested in the extent to which transplant could improve liver function in the short term, which would be critical for averting immediate organ transplant, we erred toward over-immune suppression in these early studies. Recipient monkeys were treated with Thymoglobulin and anti-CD154 at the time of transplantation and FK506 was to be used for maintenance immune suppression. All animals developed complications from over-immune suppression. Two of the three animals developed FK506 toxicity resulting from an inability to metabolize the drug (Supplementary Results).

**FIGURE 3:**
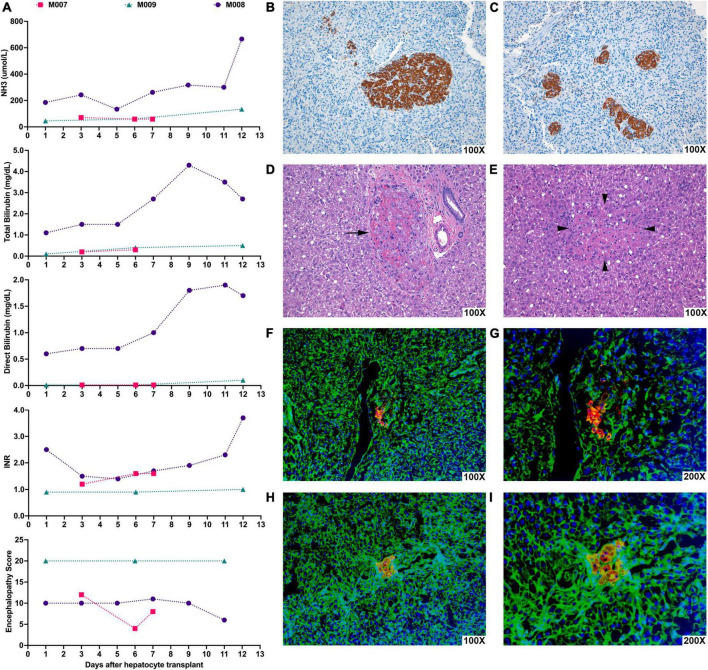
Hepatocyte xenotransplantation into the spleen of NHPs with ALF appeared to show possible transient stabilization of hepatic function and led to engraftment of donor porcine hepatocytes in the recipient spleen and liver. NHPs underwent liver-directed radiation therapy and, after 2 weeks, were subjected to 90 min of hepatic I-R injury to induce ALF. At various timepoints after the induction of ALF, NHPs were transplanted with porcine hepatocytes. **(A)** Graphs show the changes in NH3, total bilirubin, direct bilirubin, INR, and encephalopathy scores in NHPs days after porcine hepatocyte transplant (day 0). M007 underwent I-R injury and hepatocyte transplantation on day 0 and was euthanized on day 7 due to renal failure. M008 received hepatocyte transplantation 83 days after hepatic I-R injury and survived for 12 days following the transplant but eventually expired from hepatic failure. M009 received hepatocyte transplantation 14 days after hepatic I-R injury and survived for 12 days but eventually succumbed due to respiratory distress from CMV pneumonitis. **(B,C)** Representative image of immunohistochemical staining for Hep Par-1 (brown) of recipient NHP spleen showing clusters of transplanted hepatocytes in the splenic parenchyma. **(D)** Representative photomicrograph of H&E stained recipient NHP liver showing a small group of donor hepatocytes within portal veins (arrows). **(E)** Representative photomicrograph of H&E stained recipient NHP liver showing a small group of donor hepatocytes (surrounded by arrowheads) in the middle of the liver tissue. **(F–I)** Representative images of immunofluorescence staining for porcine albumin (red) and actin (green) in the recipient NHP liver showing clusters of porcine hepatocytes near or within the portal veins. Nuclei are stained with Hoechst (blue). **(G,I)** Are higher magnification images of **(F,H)**.

After transplant, we followed ammonia levels, total and direct bilirubin levels, INR, and encephalopathy scores in recipients, which did not change significantly compared to pre-transplant levels (Figure 3A). While microscopically, donor hepatocytes were found engrafted in the spleen and in the liver (Figures 3B–I), the level of engraftment was not large as there was little time for their numbers to expand even though there existed a repopulation advantage to the transplanted cells.

## Discussion

Herein, we describe the first reliable and reproducible model of ALF in NHPs. Sequential treatment of monkeys with liver directed RT and ischemia-reperfusion led reliably to acute injury and transient recovery followed by progression to ALF. Importantly, the model generated relatively uniform pathophysiology with kinetics sufficiently predictable to allow testing a new approach to therapy. The model thus appears suitable for testing a range of therapies for the management of hepatic failure. Furthermore, the model could be modified to generate an ALF model for other large animals such as pigs.

The work was developed as part of an effort to determine whether hepatocyte xenotransplantation could effectively treat acute liver failure. While our efforts to use hepatocyte transplantation to overcome the pathophysiology of acute liver failure were unsuccessful, the model allows further experimentation to definitively determine whether this approach or others can be successfully used for liver support.

Our group had previously published the effect of different doses of RT (30, 36, 40, and 50 Gy) on the liver of NHPs (17). In that study, animals treated with 30–36 Gy did not develop hepatic dysfunction from hypofractionated RT. Histological analysis of the livers showed injury to hepatic central veins, most severe in small venules, with significant extension to larger central veins at higher radiation dosage. At 30–36 Gy, central veins showed only mild medial fibrosis, and occasional prominent endothelial cells, but not intimal swelling. There were a few subendothelial inflammatory cells but the lumens were not narrowed or occluded. Interestingly, animals that received ≥ 36 Gy developed fulminant hepatic failure with encephalopathy when they received parenteral nutrition containing 15–20% dextrose. This strategy reliably generated ALF with histology and clinical manifestations approximating what is seen in the clinical setting. However, that model proved challenging to use for testing the extent to which hepatocyte transplantation could affect liver failure, as the time to induction of ALF was not predictable. For our purpose, we needed a model in which we could control both the severity and the kinetics of the disease process. Therefore, we modified the liver injury component of our model to using a known acute toxin, carbon tetrachloride (CCl_4_). We attempted to establish the susceptibility of each monkey to CCl_4_ to allow more uniform responses to the toxin. Unfortunately, we found extensive variability in the extent of acute hepatocyte injury and idiosyncratic reactions to CCl_4_.

Given the lack of a uniform response with CCl_4_, we then tested whether ischemia-reperfusion superimposed on irradiation could generate a more homogenous response. Using transient ischemia of the liver by cross-clamping the portal triad, we successfully induced reproducible hepatocyte injury following radiation conditioning of the liver and all the animals developed lab indicators of hepatic dysfunction within 1–2 weeks before succumbing to irreversible hepatic failure. Having a model that allows intervention days to weeks before death from ALF should now, hopefully impact the development of therapies for ALF. Furthermore, having parameters that anticipate death from ALF up to 14 days before the need for euthanasia allows interventions late in the course of the disease.

In severely afflicted patients with acute liver failure, the native liver can regenerate if the patient is supported for months using auxiliary liver transplant (26). Therefore, orthotopic liver transplantation might be avoided in some, perhaps many of those now transplanted for ALF (14). We, therefore, attempted to address whether hepatocyte xenografts could provide hepatic support in acute liver failure. We have previously shown that discordant hepatocyte xenografts can provide physiological hepatic function and rescue rodents with liver failure (22–24), and have shown that porcine hepatocyte xenografts can survive for many months in NHPs and expand in number in radiation conditioned livers (27, 28). However, we were not able to determine unequivocally physiologic function by those transplanted porcine hepatocyte in NHPs.

Although clinical hepatocyte transplants are performed by infusion into the portal vein (29–33), in these studies we introduced hepatocytes into the spleen to avoid portal vein stasis and portal-hepatic vein shunting. In preliminary studies, we showed that injection into spleen parenchyma led to far superior initial engraftment after radiation conditioning and no translocation of hepatocytes into the lungs, which occurred with transplantation *via* the portal vein after induction of ALF (data not shown).

Unfortunately, we did not demonstrate significant improvement in liver function in our animals with liver failure. Clearly, over-immune suppression led to the undesired complications of overwhelming opportunist infection by CMV in one recipient and FK506 toxicity in at least one other. More importantly, we found limited initial engraftment in the livers and spleens, making it unlikely that significant hepatic support was provided using the transplant techniques we employed. In previous studies of porcine hepatocyte transplant in NHPs, we demonstrated replacement of 10–15% of hepatic mass (28). However, this occurred because the donor porcine hepatocytes also had a selective repopulation advantage over native hepatocytes, but the process transpired over a period of weeks. Further studies using alternative engraftment strategies and less toxic immune suppression would be enlightening. While we were most interested in the extent to which cell transplantation could improve liver function in the short term, which would be critical for determining whether organ transplant could be averted, we over-immune suppressed our recipients. In severe hepatic failure, it is likely that significantly less immune suppression will be needed.

One of the hurdles to testing the efficacy of hepatocyte transplantation, liver support devices, and medical therapeutics for ALF has been the extraordinary variability of presentation and course of the disease. Initial studies of such devices in the laboratory are often promising because they attempt to correct hypothetical causes and outcomes of decompensated hepatic function. This is not surprising as indications for hepatic support have expanded over the years to include patients with acute liver failure, patients deteriorating from progressive end-stage chronic liver disease or alcoholic hepatitis, and finally, patients with post-hepatectomy or post-transplant liver dysfunction (14). In addition, patients with stable chronic liver disease can develop severe and rapid deterioration of liver function, termed acute-on-chronic liver failure (ACLF), from processes such as sepsis, dehydration, or variceal bleeding (34). Thus, the most crucial obstacle to developing and optimizing liver support technologies has been the lack of a reliable, quantitative, and reproducible preclinical model of acute and semi-acute liver failure for testing efficacy and toxicity. Because there is no good model of hepatic failure (except toxin-induced hepatic decompensation) and the actual mechanisms by which hepatocytes fail and the consequences of their failure are not fully understood, liver assist devices are frequently found to be ineffective upon translation out of the laboratory. The need for an ideal liver assist device is very high. We are hopeful that the model described here may be helpful for studies aimed at developing systems for temporary hepatic support.

## Data availability statement

The raw data supporting the conclusions of this article will be made available by the authors, without undue reservation.

## Ethics statement

The animal study was reviewed and approved by the University of Pittsburgh Institutional Animal Care and Use Committee.

## Author contributions

AS-G, SN, MC, JP, and IF designed the NHP model and transplant experiments. RK, NA-S, JS, JC, and IF were involved in daily management and surgery of NHPs. AOl, MQ, and MD performed CT planning and delivery of radiation therapy. BA and SN prepared and shipped porcine hepatocytes to Pittsburgh. AOs isolated donor hepatocytes and prepared the cells on arrival in Pittsburgh. GS-G and HJ performed the serum cytokine analysis. ET and MR-M performed histology and immunocytochemistry. RK, ET, JP, and IF wrote the manuscript. All authors reviewed, critiqued, and offered comments to the text.

## Abbreviations

ALF: acute liver failure
RT: radiation therapy
NHP: non-human primates
MELD: model for end-stage liver disease
I-R injury: ischemia-reperfusion injury
CT: computed tomography
CBCT: cone-beam computed radiotherapy
SBRT: stereotactic body radiotherapy treatments
CCl_4_: carbon tetrachloride
ALT: alanine aminotransferase
EN score: encephalopathy score
TPN: total parenteral nutrition
NH3: ammonia level
INR: international normalized ratio.

## References

[1] Trefts E Gannon M Wasserman DH. The liver. *Curr Biol.* (2017) 27:R1147–51. 10.1016/j.cub.2017.09.019 29112863 PMC5897118

[2] Bernal W Auzinger G Dhawan A Wendon J. Acute liver failure. *Lancet.* (2010) 376:190–201. 10.1016/S0140-6736(10)60274-7 20638564

[3] Bower WA Johns M Margolis HS Williams IT Bell BP. Population-based surveillance for acute liver failure. *Am J Gastroenterol.* (2007) 102:2459–63. 10.1111/j.1572-0241.2007.01388.x 17608778

[4] Peng Y Qi X Guo X. Child-Pugh versus meld score for the assessment of prognosis in liver cirrhosis: a systematic review and meta-analysis of observational studies. *Medicine (Baltimore).* (2016) 95:e2877. 10.1097/MD.0000000000002877 26937922 PMC4779019

[5] Cholongitas E Theocharidou E Vasianopoulou P Betrosian A Shaw S Patch D Comparison of the sequential organ failure assessment score with the king’s college hospital criteria and the model for end-stage liver disease score for the prognosis of acetaminophen-induced acute liver failure. *Liver Transpl.* (2012) 18:405–12. 10.1002/lt.23370 22213443

[6] Lock JF Kotobi AN Malinowski M Schulz A Jara M Neuhaus P Predicting the prognosis in acute liver failure: results from a retrospective pilot study using the limax test. *Ann Hepatol.* (2013) 12:556–62. 23813133

[7] Lu BR Zhang S Narkewicz MR Belle SH Squires RH Sokol RJ Evaluation of the liver injury unit scoring system to predict survival in a multinational study of pediatric acute liver failure. *J Pediatr.* (2013) 162:1010-6.e1–4. 10.1016/j.jpeds.2012.11.021 23260095 PMC3786160

[8] Bernal W Hyyrylainen A Gera A Audimoolam VK McPhail MJ Auzinger G Lessons from look-back in acute liver failure? A single centre experience of 3300 patients. *J Hepatol.* (2013) 59:74–80. 10.1016/j.jhep.2013.02.010 23439263

[9] Lee WM Squires RH Jr. Nyberg SL Doo E Hoofnagle JH. Acute liver failure: summary of a workshop. *Hepatology.* (2008) 47:1401–15. 10.1002/hep.22177 18318440 PMC3381946

[10] Bertani H Gelmini R Del Buono MG De Maria N Girardis M Solfrini V Literature overview on artificial liver support in fulminant hepatic failure: a methodological approach. *Int J Artif Organs.* (2002) 25:903–10. 10.1177/039139880202501002 12456029

[11] Kjaergard LL Liu J Als-Nielsen B Gluud C. Artificial and bioartificial support systems for acute and acute-on-chronic liver failure: a systematic review. *JAMA.* (2003) 289:217–22. 10.1001/jama.289.2.217 12517233

[12] Malchesky PS. Nonbiological liver support: historic overview. *Artif Organs.* (1994) 18:342–7. 10.1111/j.1525-1594.1994.tb02214.x 8037607

[13] O’Grady JG Gimson AE O’Brien CJ Pucknell A Hughes RD Williams R. Controlled trials of charcoal hemoperfusion and prognostic factors in fulminant hepatic failure. *Gastroenterology.* (1988) 94(Pt 1):1186–92. 10.1016/0016-5085(88)90011-x 3280388

[14] Garcia Martinez JJ Bendjelid K. Artificial liver support systems: what is new over the last decade? *Ann Intensive Care.* (2018) 8:109. 10.1186/s13613-018-0453-z 30443736 PMC6238018

[15] Selden C Bundy J Erro E Puschmann E Miller M Kahn D A clinical-scale bioartificial liver, developed for GMP, improved clinical parameters of liver function in porcine liver failure. *Sci Rep.* (2017) 7:14518. 10.1038/s41598-017-15021-4 29109530 PMC5674071

[16] Newsome PN Plevris JN Nelson LJ Hayes PC. Animal models of fulminant hepatic failure: a critical evaluation. *Liver Transpl.* (2000) 6:21–31. 10.1002/lt.500060110 10648574

[17] Yannam GR Han B Setoyama K Yamamoto T Ito R Brooks JM A nonhuman primate model of human radiation-induced venocclusive liver disease and hepatocyte injury. *Int J Radiat Oncol Biol Phys.* (2014) 88:404–11. 10.1016/j.ijrobp.2013.10.037 24315566 PMC3905315

[18] McGill MR Jaeschke H. Animal models of drug-induced liver injury. *Biochim Biophys Acta Mol Basis Dis.* (2019) 1865:1031–9. 10.1016/j.bbadis.2018.08.037 31007174 PMC6478394

[19] Li J Zhu X Liu F Cai P Sanders C Lee WM Cytokine and autoantibody patterns in acute liver failure. *J Immunotoxicol.* (2010) 7:157–64. 10.3109/15476910903501748 20039781 PMC4937798

[20] Azhar N Ziraldo C Barclay D Rudnick DA Squires RH Vodovotz Y Analysis of serum inflammatory mediators identifies unique dynamic networks associated with death and spontaneous survival in pediatric acute liver failure. *PLoS One.* (2013) 8:e78202. 10.1371/journal.pone.0078202 24244295 PMC3823926

[21] Bonkovsky HL Barnhart HX Foureau DM Steuerwald N Lee WM Gu J Cytokine profiles in acute liver injury-results from the US drug-induced liver injury network (Dilin) and the acute liver failure study group. *PLoS One.* (2018) 13:e0206389. 10.1371/journal.pone.0206389 30359443 PMC6201986

[22] Nagata H Ito M Cai J Edge AS Platt JL Fox IJ. Treatment of cirrhosis and liver failure in rats by hepatocyte xenotransplantation. *Gastroenterology.* (2003) 124:422–31. 10.1053/gast.2003.50065 12557148

[23] Minato M Houssin D Demma I Morin J Gigou M Szekely AM Transplantation of hepatocytes for treatment of surgically induced acute hepatic failure in the rat. *Eur Surg Res.* (1984) 16:162–9. 10.1159/000128404 6373295

[24] Sutherland DE Numata M Matas AJ Simmons RL Najarian JS. Hepatocellular transplantation in acute liver failure. *Surgery.* (1977) 82:124–32. 327598

[25] Nagata H Nishitai R Shirota C Zhang JL Koch CA Cai J Prolonged survival of porcine hepatocytes in cynomolgus monkeys. *Gastroenterology.* (2007) 132:321–9. 10.1053/j.gastro.2006.10.013 17241882

[26] Chenard-Neu MP Boudjema K Bernuau J Degott C Belghiti J Cherqui D Auxiliary liver transplantation: regeneration of the native liver and outcome in 30 patients with fulminant hepatic failure–a multicenter european study. *Hepatology.* (1996) 23:1119–27. 10.1002/hep.510230528 8621143

[27] Yamanouchi K Zhou H Roy-Chowdhury N Macaluso F Liu L Yamamoto T Hepatic irradiation augments engraftment of donor cells following hepatocyte transplantation. *Hepatology.* (2009) 49:258–67. 10.1002/hep.22573 19003915 PMC3416044

[28] Soltys KA Setoyama K Tafaleng EN Soto Gutierrez A Fong J Fukumitsu K Host conditioning and rejection monitoring in hepatocyte transplantation in humans. *J Hepatol.* (2017) 66:987–1000. 10.1016/j.jhep.2016.12.017 28027971 PMC5395353

[29] Anderson TN Zarrinpar A. Hepatocyte transplantation: past efforts, current technology, and future expansion of therapeutic potential. *J Surg Res.* (2018) 226:48–55. 10.1016/j.jss.2018.01.031 29661288 PMC6558656

[30] Dagher I Boudechiche L Branger J Coulomb-Lhermine A Parouchev A Sentilhes L Efficient hepatocyte engraftment in a nonhuman primate model after partial portal vein embolization. *Transplantation.* (2006) 82:1067–73. 10.1097/01.tp.0000236103.99456.8f 17060856

[31] Gaillard M Dagher I. Minimally invasive liver preconditioning for hepatocyte transplantation in rats. *Methods Mol Biol.* (2017) 1506:193–200. 10.1007/978-1-4939-6506-9_13 27830554

[32] Kobayashi E Enosawa S Nagashima H. Experimental hepatocyte transplantation in pigs. *Methods Mol Biol.* (2017) 1506:149–60. 10.1007/978-1-4939-6506-9_10 27830551

[33] Shibuya K Watanabe M Goto R Zaitsu M Ganchiku Y Taketomi A. The efficacy of the hepatocyte spheroids for hepatocyte transplantation. *Cell Transplant.* (2021) 30:9636897211000014. 10.1177/09636897211000014 33900126 PMC8085376

[34] Alam A Chun Suen K Ma D. Acute-on-chronic liver failure: recent update. *J Biomed Res.* (2017) 31:283–300. 10.7555/JBR.30.20160060 28808200 PMC5548989

