# Supplementary material for: A non-human primate model of acute liver failure suitable for testing liver support systems
Source: Front Med (Lausanne). 2022 Sep 30;9:964448. doi: 10.3389/fmed.2022.964448 (PMC9561471; doi:10.3389/fmed.2022.964448)
Supplement: Supplementary file 1 [file Data_Sheet_1.pdf]

Supplementary Material

SUPPLEMENTARY MATERIALS AND METHODS

Hepatocyte Preparation

Hepatocytes were isolated at the Mayo Clinic from pigs weighing between 6 kg and 15 kg using collagenase perfusion methods previously described (1, 2) and stored at the animal care facility at the Mayo Clinic. Viability of isolated hepatocytes was determined by trypan blue exclusion. Initial cell viability exceeded 90%. Cells were suspended in HTS-FRS hepatocyte storage medium at a concentration of 5 million cells per mL and shipped over night on wet ice to the University of Pittsburgh. Upon arrival the porcine hepatocytes were centrifuged 4 times at 700 rpm to clear the storage medium and resuspended in saline Dulbecco's Modified Eagle medium. Cell viability (as determined by trypan blue exclusion) was 80-84%, and the plating efficiency at 24 hours was 60-90%.

Intrahepatic Liver Cell Transplantation in NHPs

In preliminary studies, portal infusion of hepatocytes was performed in animals pre-treated with liver directed radiation through a midline incision. The small bowel mesentery was brought into the field and a 20-gauge angiocath was inserted into a mesenteric vessel, and a 0.018 guide wire was advanced through the angiocath into the main portal vein. The angiocath was then replaced with a 4f intravascular sheath and a portal venogram was obtained via the sheath to determine the vascular anatomy. Maintenance of portal flow integrity was confirmed every 15 minutes during the infusion by performing contrast portal venography to identify shunting or flow stasis of the portal circulation. Fresh porcine hepatocytes in Dulbecco's Modified Eagle medium were injected through the portal vein catheter. The catheter was then removed and mesenteric vessel bleeding was controlled with ligatures of 3-0 silk.

Intrasplenic Liver Cell Transplantation in NHPs

Intrasplenic liver cell transplant was performed using either open surgery or using ultrasound-guided percutaneous injection. Porcine hepatocytes, suspended as a packed volume of 15-30 mL, were injected directly into the parenchyma of the spleen using an 18-gauge needle over approximately 20 minutes. The needle was redirected into different portions of the splenic parenchyma to distribute the cells more completely. Hemostasis was obtained by electrocautery if an open approach was elected. If a percutaneous route of administration was chosen, hemostasis was ensured via injection of a gel foam slurry. No peri-splenic hematoma was identified by ultrasound or at autopsy following ultrasound-guided transplantation. Whether cells are introduced directly into the portal circulation or into the spleen, the cells can translocate out of the initial implantation site and home to the liver.

Immune Suppression and Antiviral Therapy

The level of immune suppression needed to control hepatocyte xenograft rejection while the recipient is in liver failure is unknown. Since our major concern was initial engraftment and determining function that might defer the need for immediate organ transplantation, recipients were given induction therapy that included Thymoglobulin®, a rabbit anti-thymocyte antibody (Genzyme Transplant, Cambridge, MA; 4 mg/kg IV) on days -1 to day +2 and the anti-CD154 clone 5C8 mouse human chimeric antibody (NHP Reagent Resource, MassBiologics of the University of Massachusetts Medical School, Boston, MA; 25 mg/kg IV) on days -1, 0, and +4 with respect to transplant. FK506/Tacrolimus (Astellas Pharma USA, Deerfield, IL; 0.05 or 0.01 mg/kg SC) was given at various times after transplant to maintain blood levels of 10-15 ng/mL. Ganciclovir was given at 5 mg/kg BID for 2 weeks in animals suspected of developing systemic CMV infection based on low WBC and clinical suspicion (decreased activity and appetite).

Immunohistochemistry and Immunofluorescence Staining

Details of antibodies and reagents are listed in **Supplementary Table 2**. For immunohistochemistry, tissue samples were fixed in 4% paraformaldehyde (PFA) in phosphate buffered saline (PBS) for 24 hours

at 4°C, incubated in 70% ethanol for 24 hours at 4°C, and paraffin-embedded. Tissue immunohistochemistry was performed on the Ventana Benchmark Ultra IHC automated staining platform (Roche Diagnostics, Indianapolis, IN). Samples incubated with Hep Par-1 antibody were visualized using the Ventana iView DAB detection kit. Samples incubated with anti-cytomegalovirus antibody were visualized using Ventana Ultraview Universal DAB detection kit. For immunofluorescence staining, tissue samples were fixed in 2% PFA in PBS for 24 hours at 4°C, immersed in 30% sucrose in PBS for 24 hours at 4°C, and then frozen in Tissue-Tek O.C.T. compound (VWR, Philadelphia, PA). Frozen tissue samples were stored at -80°C until cryosectioning. Five-micron cryosections were washed 3x with PBS and then 3x with wash buffer (0.1% bovine serum albumin (BSA)/0.1% Tween 20 in PBS). Sections were incubated with blocking buffer (10% normal donkey serum/1% BSA/0.1% Tween20/0.1% Triton X-100 in PBS) at room temperature for 1 hour and incubated with anti-porcine albumin in blocking buffer overnight at 4°C. Slides were washed 3x with wash buffer and incubated with secondary antibody and phalloidin-AF488 in blocking buffer at room temperature for 1 hour in the dark. Slides were washed 3x with wash buffer and nuclei were then stained for 2 minutes with Hoechst. After washing 3x with PBS, sections were mounted using Fluoroshield (Sigma-Aldrich, St. Louis, MO). Samples were imaged using an Eclipse Ti inverted microscope (Nikon Instruments Inc., Melville, NY) and the NIS-Elements software platform (Nikon Instruments Inc., Melville, NY).

SUPPLEMENTARY RESULTS

Hepatocyte Xenotransplantation in NHPs with ALF

M007 underwent I-R induced liver injury and transplantation on the same day **(Table 1** and **Figure 3)**. He received 300 million porcine hepatocytes directed into the spleen during his laparotomy. After transplant, liver function appeared to stabilize briefly. However, he was euthanized 7 days after transplant with renal failure. FK506 (0.05 mg/kg) was given peri-operatively. However, because of his hepatic ischemia-reperfusion injury, he was not able to metabolize FK506 and after 2 doses he developed FK506 toxicity

and never cleared the medication. As a result, dosing of FK506 was modified for other transplanted animals.

M008 was transplanted using an open technique with 600 million cells once liver failure developed after radiation and hepatic I-R injury **(Table 1** and **Figure 3)**. FK506 therapy was withheld until post-transplant day 7. He was only given one FK506 dose which was one-fifth of the starting dose given to M007. He still had a toxic level of 42.9 ng/mL 24 hours after receiving his dose but the FK506 level came down to 16.4 ng/mL on post-transplant day 12. After transplant, he had a relatively stable ammonia level and encephalopathy score until the day of his death when his ammonia level rose from 300 to 600 umol/L and his encephalopathy score crashed. He died 12 days after transplant from hepatic failure **(Figure 3)**. Because reactivation of CMV is common in our immune suppressed NHPs, he was treated with prophylactic ganciclovir. Post mortem analysis of tissues identified CMV inclusions in the spleen but not in other organs, but immunohistochemistry for CMV was negative throughout, indicating no active CMV infection.

M009 was transplanted 2 weeks after radiation and hepatic I-R injury **(Table 1** and **Figure 3)**. He received one billion hepatocytes into his spleen via ultrasound guided percutaneous injection. He achieved therapeutic levels of FK506 throughout his course and appeared stable during transplant. After transplant, liver function appeared to stabilize briefly **(Figure 3)**. However, he developed respiratory distress on post-transplant day 14 and expired from CMV pneumonitis confirmed at autopsy. At autopsy he had severe diffuse interstitial pneumonia with intervascular thrombi. CMV inclusions were also found in both the lungs and the liver, possibly explaining the deterioration in liver function.

SUPPLEMENTARY REFERENCES

1. Nyberg SL, Hardin J, Amiot B, Argikar UA, Remmel RP, Rinaldo P. Rapid, Large-Scale Formation of Porcine Hepatocyte Spheroids in a Novel Spheroid Reservoir Bioartificial Liver. Liver Transpl (2005) 11(8):901-10. Epub 2005/07/22. doi: 10.1002/lt.20446.

2. Sielaff TD, Hu MY, Rao S, Groehler K, Olson D, Mann HJ, et al. A Technique for Porcine Hepatocyte Harvest and Description of Differentiated Metabolic Functions in Static Culture. Transplantation (1995) 59(10):1459-63. Epub 1995/05/27. doi: 10.1097/00007890-199505270-00017.

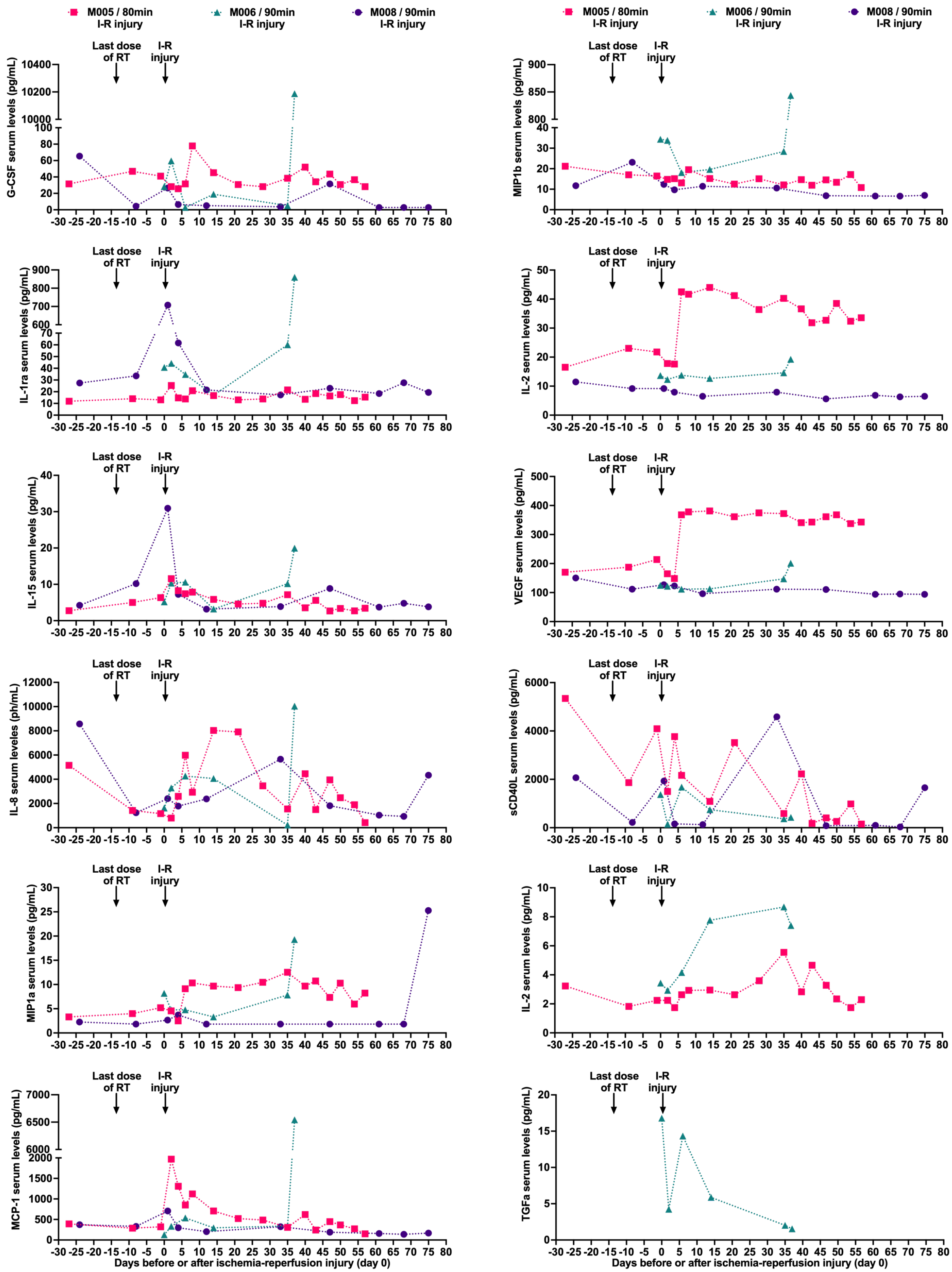
M005 / 80min I-R injury
M006 / 90min I-R injury
M008 / 90min I-R injury
Last dose of RT
I-R injury
G-CSF serum levels (pg/mL)
MIP1b serum levels (pg/mL)
IL-1ra serum levels (pg/mL)
IL-2 serum levels (pg/mL)
IL-15 serum levels (pg/mL)
VEGF serum levels (pg/mL)
IL-8 serum leveles (ph/mL)
sCD40L serum levels (pg/mL)
MIP1a serum levels (pg/mL)
IL-2 serum levels (pg/mL)
MCP-1 serum levels (pg/mL)
TGFa serum levels (pg/mL)
-30 -25 -20 -15 -10 -5 0 5 10 15 20 25 30 35 40 45 50 55 60 65 70 75 80
Days before or after ischemia-reperfusion injury (day 0)
Days before or after ischemia-reperfusion injury (day 0)

Supplementary Figure 1. Serum cytokine levels in NHPs treated with liver-directed radiation therapy and hepatic ischemic-reperfusion injury. NHPs underwent liver-directed RT and, after 2 weeks, were subjected to 80-90 minutes of hepatic I-R injury. Serum cytokine levels were measured at several time points before and after hepatic I-R injury (day 0). Measurements were performed using the MILLIPLEX MAP Non-Human Primate Cytokine Magnetic Bead Panel - Premixed 23 Plex - Immunology Multiplex Assay, Millipore Cat: PCYTMG-40K-PX23. There was no consistent trend in the serum cytokine levels in this NHP model of ALF.

Supplementary Table 1. Encephalopathy score rating scale

	5 points	4 points	3 points	2 points	1 point
**Appetite**	No impairment in appetite. Eats all biscuits, fruits, and vegetables	No interest in biscuits but has strong appetite for fruits and vegetables	No interest in biscuits but adequate appetite for fruits and vegetables. Food consumed by the end of the day or overnight	Very little interest in food after eating a small portion. Remaining food not consumed by the end of the day or overnight	No interest in food. Refuses all biscuits, fruits, and vegetables
**Attention**	Completely attentive	Alert. Directional attention preserved. Frequently willing to participate in training	Alert. Directional attention preserved. Some willingness to participate in training	Sporadically zoned out. Seems disassociated from the situation	No interest in moving or participating in training.
**Neurologic Condition**	Strong stable grasp with no shaking	Some muscular weakness but not a level that interferes with normal function.	Impaired grasp. Requires two to three tries to forage from the bottom of the cage. Increased hand tremors	Impaired grasp. Requires more than three tries to forage from the bottom of the cage. Markedly increased hand tremors	No interest in moving or participating in training
**Strength**	Strong and stable with no muscular weakness and shaking. Normal locomotive behaviors. Ability to maintain stable position is preserved	Some muscular weakness exhibited but not at a level that interferes with normal locomotion or maintenance of stable position	Increased weakness. Impaired ability to hold stable position for more than two to three seconds without balance issues or compensation of some sort	Markedly increased weakness and instability. Difficulty in any locomotive behavior	No interest in moving or participating in training

Supplementary Table 2. Antibodies/reagents and corresponding dilutions/concentrations used for immunohistochemistry and immunofluorescence staining.

Antibody/Reagent	Host	Company	Catalogue No.	Dilution/Concentration
Hep Par-1, clone OCH1E5	mouse	Dako, Carpinteria, CA	M7158	1:500
anti-cytomegalovirus, clones CCH2 + DDG9	mouse	Dako, Carpinteria, CA	M0854	1:100
anti-porcine albumin	goat	Bethyl Labs, Montgomery, TX	A100-110A	1:500
anti-goat IgG-AFP555	donkey	Life Technologies, Carlsbad, CA	A32816	1:250
Phalloidin-AF488	N/A	Life Technologies, Carlsbad, CA	A12379	0.165 uM
Hoechst 33342	N/A	Life Technologies, Carlsbad, CA	H3570	1 ug/mL
